# Correlation of pretreatment drug induced apoptosis in ovarian cancer cells with patient survival and clinical response

**DOI:** 10.1186/1479-5876-10-162

**Published:** 2012-08-08

**Authors:** Emery Salom, Manuel Penalver, Howard Homesley, Matthew Burrell, Audrey Garrett, Cary A Presant, James Rutledge, Michael Chernick, Allan Hallquist, Mathieu Perree

**Affiliations:** 1 South Florida Gynecologic Oncology, Coral Gables, FL, USA; 2 East Carolina University Brody School of Medicine, Greenville, NC, USA; 3 Wake Forest University School of Medicine, Winston-Salem, NC, USA; 4 Northside Hospital of Atlanta, Atlanta, GA, USA; 5 Sacred Heart Medical Center, Eugene, OR, USA; 6 Data Vision, Dayton, OH, USA; 7 The Lankenau Institute of Medical Research, Wynnewood, PA, USA; 8 Wilshire Oncology Medical Group – US Oncology, Los Angeles, CA, USA; 9 DiaTech Oncology, Montreal, Canada; 10 Palmetto General Hospital, 2001 W. 68th St, Hialeah, FL 33016, USA

## Abstract

**Background:**

This study was performed to determine if a chemotherapy-induced apoptosis assay (MiCK) could predict the best therapy for patients with ovarian cancer.

**Methods:**

A prospective, multi-institutional and blinded trial of the assay was conducted in 104 evaluable ovarian cancer patients treated with chemotherapy. The MiCK assay was performed prior to therapy, but treating physicians were not told of the results and selected treatment only on clinical criteria. Outcomes (response, time to relapse, and survival) were compared to the drug-induced apoptosis observed in the assay.

**Results:**

Overall survival in primary therapy, chemotherapy naïve patients with Stage III or IV disease was longer if patients received a chemotherapy which was best in the MiCK assay, compared to shorter survival in patients who received a chemotherapy that was not the best. (p < 0.01, hazard ratio HR 0.23). Multivariate model risk ratio showed use of the best chemotherapy in the MiCK assay was the strongest predictor of overall survival (p < 0.01) in stage III or IV patients. Standard therapy with carboplatin plus paclitaxel (C + P) was not the best chemotherapy in the MiCK assay in 44% of patients. If patients received C + P and it was the best chemotherapy in the MiCK assay, they had longer survival than those patients receiving C + P when it was not the best chemotherapy in the assay (p = 0.03). Relapse-free interval in primary therapy patients was longer if patients received the best chemotherapy from the MiCK assay (p = 0.03, HR 0.52). Response rates (CR + PR) were higher if physicians used an active chemotherapy based on the MiCK assay (p = 0.03).

**Conclusion:**

The MiCK assay can predict the chemotherapy associated with better outcomes in ovarian cancer patients. This study quantifies outcome benefits on which a prospective randomized trial can be developed.

## Introduction

Epithelial ovarian cancer is responsive to several chemotherapeutic drugs. However, there is considerable variability in individual response to these drugs in combination or as single agents. Oncologists have wanted to develop an assay that can predict response to chemotherapy drugs and combinations to allow individualized cancer therapy.

The issue of predictive testing for choosing chemotherapy for cancer patients is of very high interest. The MiCK assay for drug-induced apoptosis is a non-genomic test that is in national trials in a number of different tumors.

Assays for chemotherapy resistance have been developed in the past, but their application in ovarian cancer has been of limited usefulness [1]. NCCN acknowledges that some institutions do use these tests in patients, but their general use is not guideline-approved [2].

A drug-induced apoptosis assay, the microculture-kinetic test (MiCK), has been developed [3] and tested with success in acute myelocytic leukemia [4,5]. The basis of this assay is the ability of a drug to rapidly induce apoptosis in cancer cells in short-term culture (48 hours) without a necessity for tumor cell growth. In addition to testing in AML, it has been undergoing testing in solid tumors including breast cancer [6], endometrial cancer [7], lung cancer, miscellaneous solid tumors and hematologic malignancies (the results of a non-blinded trial are in press, Cancer, 2012). These results indicate that the MiCK assay is more predictive of response and survival than almost all currently available genomic tests (probably because there are so many mutations, and epigenetic changes in every cancer, most usually different between cancers of similar histologic type, and often differing between metastases in the same patient).

We report here the results of a multi-center, prospective, non-randomized, and physician-blinded study of the MiCK assay in patients with ovarian cancer.

## Methods

Patients with epithelial ovarian cancer of any stage, primary or recurrent, were eligible. Sterile tumor specimens (minimum 250 mg but optimally with >1.0 cm^3^ of viable tumor tissue) were placed into sterile DiaTech transport media, and sent via FedEx to the DiaTech Oncology laboratory, Montreal, Canada.

### Tumor cell purification

Within 24 to 48 hours of collection, the specimen was minced, digested with 0.25% trypsin and 0.08% DNase for 1–2 hours at 37 degrees Celsius, and then filtered through a 100 micrometer cell strainer. Tumor cells were purified by a proprietary method which included density gradient centrifugation, and incubation with a series of antibody-coated beads to allow only viable tumor cells to remain. The pathologist confirmed that the remaining cells in the assay were at least 90% viable ovarian cancer cells. The final cell suspension was plated into a 96-well half-area plate, 120 microliter aliquot per well. The plate was incubated overnight at 37 degrees Celsius with 5% carbon dioxide humidified atmosphere. 5x10^4^ to 1.5x10^5^ cells were seeded per well depending on the cell volume to give adequate well-bottom coverage.

Human JURL-MK2 chronic leukemia in blast crisis cell line (DSMZ, Germany) was used as a positive control for MiCK assays performed with patient tumor cells. RPMI-1640 medium without phenol red was used for all cultures. It was supplemented with 10% fetal bovine serum, 100 units/mL of penicillin, and 100 micrograms/mL of streptomycin. Cell counts and viability were evaluated by trypan blue dye exclusion.

Each tumor cell preparation was reviewed by a pathologist using a hematoxylin/eosin stained cytospin preparation to confirm the presence of malignant cells consistent with an ovarian carcinoma primary. If there was an adequate number of cells available, immunocytochemical stains including CK, CA125, calretinin and Ki-67 were performed to add supportive evidence of an ovarian origin as well as mitotic activity. The cell suspensions were purified repetitively until at least 90% purity was obtained. Suspensions with under 90% purity were not tested in the MiCK assay.

### MiCK assay for apoptosis

The MiCK assay procedure was adapted from the method described previously [3,4]. After overnight incubation, chemotherapy drugs were added in the wells of the 96-well plate in 5 microliter aliquots at various concentrations. Single and combination regimens were tested, as seen in Table 1.

**Table 1:** Drug-Induced Apoptosis in the MiCK Assay

**Drug**	**# 0f Patients**	**Mean Assay KU**	**Minimum Assay KU**	**Maximum Assay KU**
Carboplatin	69	1.8	0	12.4
Paclitaxel	73	2.2	0	9.7
Carboplatin + Paclitaxel	99	2.9	0.7	8.2
Cisplatin	45	2.7	0	9.1
Gemcitabine	41	0.9	0	3.4
Cisplatin + Gemcitabine	82	2.5	0	11.4
Docetaxel	9	2.1	0	3.4
Topotecan	67	1.5	0	6.8
4hydroxycyclophosphamide	20	2.9	0	15.4
Doxorubicin	4	3.3	0.4	6.9
Liposomal doxorubicin	65	1.0	0	5.3
Epirubicin	9	3.2	0.4	7.7
Albumin-bound Paclitaxel	4	0.7	0	1.3

Three concentrations of each drug or combination were tested based on the distribution of standard drug dose in total body water as the mid-range concentration, with another concentration above and below the mid-range concentration. Following drug addition, the plate was incubated for 30 min at 37 degrees Celsius into a 5% carbon dioxide humidified atmosphere incubator. Each well was then overlayed with mineral oil, and the plate was placed into the incubator chamber of a microplate spectrophotometric reader (BioTek instruments). The optical density at 600 nanometers was read and recorded every 5 minutes over a period of 48 hours. Optical density increases, which correlate with apoptosis, were converted to kinetic units (KU) of apoptosis by a proprietary software ProApo with a formula described previously [3,4] and were correlated with patient outcomes. Spontaneous apoptosis was controlled for using control wells containing patient tumor cells without drugs. Active apoptosis was indicated as > 1.0 KU.

### Treatment of patients

This study was a prospective multi-institutional blinded trial. It was non-randomized, and thus was an observational study. MiCK assay results were obtained before any therapy was initiated. MiCK assay results were never transmitted to physicians. Physicians treated patients with the physicians’ own choice of drugs as they deemed clinically indicated. Tumor responses were measured by RECIST criteria every 3 months. Patients were evaluated for response, time to recurrence after assay and disease-specific survival after assay. The clinical evaluations of response, time to recurrence and survival were then compared to the in vitro MiCK assay drug-induced apoptosis that had been determined before chemotherapy was initiated.

### Statistical evaluation

The primary goal of the study was to compare MiCK assay results with overall survival in all patients studied. We also evaluated the subset of patients with chemotherapy-naïve stage III and IV ovarian cancer receiving primary therapy for overall survival, response rate, and recurrence-free survival. Data were imported into SAS/JMP (JMP Version 7 for Windows [8]) for analysis. If a sample had multiple doses of the same drug, then the dose with the highest value was assigned to the drug. SAS/JMP was used to calculate summary statistics and perform statistical analysis (see Table 2). Comparisons between drugs were made by subtracting one drug’s KU value from another drug’s KU value on a matched sample basis. The nonparametric Wilcoxon test [9] was then used to test for a statistical difference. All tests were two-tailed. Multivariate analysis was performed using the Cox proportional hazards model [10]. In order to account for multiplicity, MULTTEST (SAS 9.2) was used to compute adjusted p-values by the Hochberg, Holm and false discovery rate methods [11-13]. Statistically significant results remained significant at the 0.05 level.

**Table 2:** Multivariate Analysis of Overall Survival

**Term**	**Risk Ratio**	**Lower CL**	**Upper CL**	**p-value**
Age	0.86	0.05	13.57	0.92
Debulking (sub = 0 optimal = 1)	0.36	0.07	1.72	0.19
Drug (non Best = 0 Best = 1)	0.21	0.05	0.65	< 0.01
Ln ca 125	1.18	0.08	19.29	0.90
Size of residual (none = 0 some = 1))	0.59	0.12	2.24	0.45

Cox proportional hazards model for 61 evaluable patients with stage III or IV primary disease. Risk ratios calculated per change in regressor over entire range. Patients (n = 3) with no debulking were omitted. CL, confidence limit of risk ratio.

### Definition of best chemotherapy

The best chemotherapy was defined as any single drug or drug combination with the highest KU +/− 0.57 KU in an individual patient’s MiCK assay. Several different drugs or combinations could all be considered a best chemotherapy if they were all within 0.57 KU of each other. The 0.57 KU cut-off was based on the standard deviation 0.57 KU using analysis of variance in tests of replicate tumor samples in the MiCK assay.

### Investigational review board approval

IRB approval was obtained and monitored by the Western IRB in Seattle, Washington. Each patient had given voluntary informed consent in writing prior to submission of tumor specimen for MiCK analysis. The trial was registered at clinicaltrials.gov at study NCT00531388.

## Results

### Patients studied

Specimens were submitted from 210 patients between May, 2006 and September, 2010. Of these, 60 were unsuccessful due to insufficient number of viable cancer cells (40% of the 60), spontaneous necrosis in transit (17%), or delays in transit. Recent experience since 2009 has demonstrated over 75% successful cultures as these problems have been resolved by better instructions to surgeons and pathologists.

The remaining 150 patients had tumor submitted and analyzed, and were evaluable for patterns of in vitro assay of drug effects on ovarian cancer cells (see results in Table 1). Of those 150 patients, 17 patients had no outcome data since they did not return after discharge postoperatively, despite extensive efforts to obtain that data. Nearly all of those patients also declined postoperative chemotherapy. 29 of the remaining 133 patients either did not receive chemotherapy (24 patients), received chemotherapy for which there is no assay (3 patients receiving EC145 on a clinical trial, bleomycin, or rituximab plus CHOP), or had therapy that could not be matched to the MiCK assay results because of too few assays (2 patients), leaving 104 patients who received chemotherapy and were evaluable for clinical correlations with assay results.

**Figure 1:**
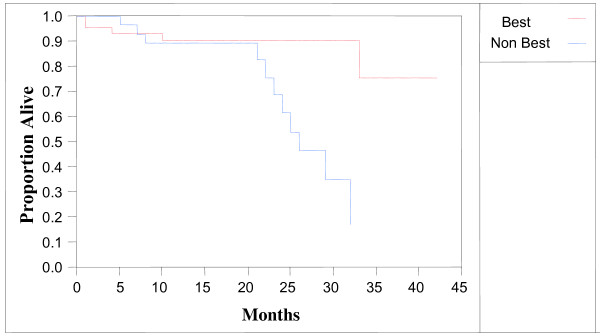
**Overall survival of patients with stage 3 or 4 primary disease without prior chemotherapy.** Red line, 44 patients receiving a best chemotherapy. Blue line, 29 patients receiving non-best chemotherapy. P < 0.01.

Of the 104 patients who received chemotherapy, 77 were stages III or IV primary treatment patients. Table 3 describes the patient characteristics of all 104 patients and those with chemotherapy-naïve stage III or IV cancer. The tumor histologies of all patients and of the stage III-IV patients, respectively, were papillary-serous 66/48; ovarian adenocarcinoma (not otherwise specified) 23/22; endometroid 6/2; clear cell 2/2; mucinous 1/1; and mixed + combined + other 6/2. Of the 104 patients, 98 had documented survival data and are described in Figure 1.

**Table 3:** Patient Characteristics

	**All**		**Primary Stage III & IV**	
	**Patients**	**%**	**Patients**	**%**
Number of patients	104	100	77	100
Stage				
I	5	4.8 %		
II	2	1.9 %		
III	67	64.4 %	67	87.0 %
IV	10	9.6 %	10	13.0 %
Recurrent	20	19.2 %		
Debulking				
No	5	4.8 %	3	3.9 %
yes optimal	79	76.0 %	58	75.3 %
yes suboptimal	20	19.2 %	16	20.8 %
Size of residual				
0	56	56.6 %	39	52.7 %
<1 cm	18	18.2 %	15	20.3 %
>1 cm	25	25.3 %	20	27.0 %
Lines of therapy before MiCK				
0	87	83.7 %	77	100 %
1	4	3.8 %		
2	3	2.9 %		
3	10	9.6 %		
Preoperative CA125				
mean	1714.5		2147.6	
median	384.5		510.5	
range	4 - 26,728		4 - 26,728	
missing	13		11	
Age				
mean	59.4		59.7	
median	60		60	
range	35 - 86		37 - 86	

### In vitro apoptosis results

Table 1 lists the results of testing with each of the drugs and/or combinations in 150 patients. Since the yield of tumor cells varied, not all patients had each drug and combination tested. The most active compounds included the combination of carboplatin and paclitaxel, and the single agents 4-hydroxy-cyclophosphamide (the activated form of cyclophosphamide), epirubicin, docetaxel, and cisplatin.

Analyses compared the results in individual patients using two drug combinations of carboplatin plus paclitaxel versus carboplatin as a single drug, and versus paclitaxel as a single drug (n = 103). In the entire patient study, combination of carboplatin plus paclitaxel was significantly more effective in producing apoptosis than carboplatin alone (p < 0.0001) or paclitaxel alone (p < 0.0001). However, in individual patient tests, compared to carboplatin plus paclitaxel, either carboplatin or paclitaxel as single drugs gave more apoptosis than the combination in 25 out of 90 evaluations (28%), and single drugs gave results equal to the combination in 8 out of 90 patients (9%).

Also, the results of cisplatin plus gemcitabine were compared to cisplatin as a single drug or gemcitabine as a single drug (n = 55). The combination of cisplatin plus gemcitabine was significantly better in producing apoptosis overall compared to gemcitabine alone (p < 0.0001). In individual patient assays, however, compared to the combination of cisplatin plus gemcitabine, the single drugs gave more apoptosis than the combination in 14 out of 44 patients (32%) and the single drugs produced apoptosis equal to the combination in 8 out of 44 patients (18%). No patients were treated clinically with these single agents.

### Correlations of MiCK apoptosis results with overall survival in all patients studied

Because all ovarian cancer patients were eligible per protocol for MiCK assay, this section presents the results in all patients studied. The overall survival of all 98 evaluable patients treated with chemotherapy was compared to the results of the MiCK apoptosis assay. In this comparison, the 60 patients who were treated with the best chemotherapy in the MiCK assay had a superior overall survival compared to the 38 patients who received a non-best chemotherapy (p = 0.003 by log rank analysis). Median survival was over 45 months for patients who received the best chemotherapy compared to 25 months for patients who received a non-best chemotherapy. The primary goal of the study was met. In addition, of the patients treated with the best chemotherapy, 53.3% recurred, compared to 76.3% recurrence in patients who received a non-best chemotherapy (p = 0.02).

### Evaluation of patients with chemotherapy-naïve stages III or IV primary therapy

#### Overall survival

This section presents the results in the more homogeneous subset of patients, those with chemotherapy-naïve stages III or IV most of whom had received tumor debulking (Table 3). No patient had received neoadjuvant therapy. The overall survival of all stages III or IV patients with primary disease was 51.2% +/− 10.7% at 42 months (this is comparable to the results of GOG protocol 182 [14], GOG protocol 218 [15], and AGO-OVAR 9 [16]).

In the chemotherapy-naïve stage III or IV ovarian cancer patients (73 with fully evaluable survival data), patients treated with the best chemotherapy in the MiCK assay had a superior survival compared to patients treated with a non-best chemotherapy (Figure 1, p < 0.01). The hazard ratio for death in patients receiving the best chemotherapy was 0.23 (95% confidence interval 0.06 to 0.67). Median survival was over 45 months for patients who received the best chemotherapy compared to 25 months for patients who received a non-best chemotherapy.

In order to exclude the possibility that there was an accidental selection of more favorable patients into pre the best chemotherapy group, a multivariate analysis was performed in the stage III and IV patients with primary therapy who had complete data to determine if the MiCK assay results were predictive of survival compared to other prognostic variables in prior analyses (Table 2). The multivariate Cox proportional hazard model showed that use of the best chemotherapy in the MiCK assay was the strongest predictor of overall survival. The estimated hazard risk ratio (relative risk) for death if a patient received the best chemotherapy was 0.21 (95% confidence interval 0.05 to 0.65, p < 0.01). Although prior studies have indicated that optimal debulking and having less residual disease were favorable prognostic factors, those factors were less prognostic when the MiCK assay results were considered in the same analysis.

In order to confirm that the MiCK assay was predictive in patients with the most optimal prognostic characteristics, we analyzed patients with stage III and IV disease, no prior chemotherapy, optimally debulked with no residual disease. Of those patients, 24 were treated with the best therapy as analyzed in the MiCK assay, and 14 were treated with drugs that were not the best in the assay. Median survival I those treated with the best drugs was over 42 months, compared to median survival of only 25 months in those who received chemotherapy that was not the best (p < 0.01).

### Recurrence-free interval

**Figure 2:**
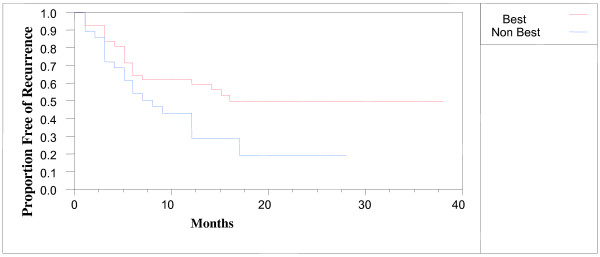
**Recurrence-free interval of patients with stage 3 or 4 primary disease without prior chemotherapy.** Red line, 43 patients receiving a best chemotherapy. Blue line, 29 patients receiving non-best chemotherapy. P = 0.03.

In the 72 patients with fully evaluable recurrence data who had chemotherapy-naïve stage III or IV primary disease, the recurrence-free interval of patients treated with the best chemotherapy was longer compared to patients treated with a non-best chemotherapy (Figure 2, p = 0.03). The hazard ratio for recurrence in patients receiving the best chemotherapy was 0.52 (95% confidence interval 0.28 to 0.96). Median recurrence-free interval was 16 months in patients receiving the best chemotherapy versus 6 months for patients receiving a non-best chemotherapy.

A multivariate analysis was performed in these patients with complete data to determine if the MiCK assay results were predictive of recurrence-free interval. Use of the best chemotherapy in the MiCK assay gave a hazard ratio for recurrence of 0.60, but this was of only marginal statistical significance (95% confidence interval 0.31 to 1.16, p = 0.13).

### Correlations of MiCK apoptosis results with response

We next evaluated whether or not there was a relationship between apoptosis in the assay compared to clinical response. In patients with chemotherapy-naïve stage III or IV primary disease, if the physicians used chemotherapy that had high apoptosis > 2 KU, 90% of the patients had a complete or partial response, compared to 74% response if the physicians used chemotherapy that had apoptosis </= 2 KU (p =0.10). If patients had received chemotherapy with any apoptosis >1.14 KU, 87% had complete or partial response, compared to only 40.0% of patients who received less active chemotherapy </= 1.14 KU (p = 0.03). The overall response rate for all stage III or IV patients was 83.3%.

### Can the MiCK assay improve standard therapy with carboplatin plus pactlitaxel?

In the 51 patients with stage III or IV disease who received standard therapy with carboplatin + paclitaxel (C + P), we studied whether C + P was best therapy in the MiCK assay. In 28 of 51 (56%), C + P was the best chemotherapy based on the assay. It was not the best chemotherapy in 44%, and the most usual better choice by the MiCK assay was gemcitabine plus cisplatin in 10 (43%). When physicians used C + P, time to recurrence was significantly longer if it was the best therapy compared to patients in whom it was a non-best therapy (p = 0.03); and overall survival was marginally longer if C + P was the best therapy versus if C + P was a non-best therapy (p = 0.1) with 88% survival at 30 months if C + P was the best therapy, versus 20% if it was a non-best therapy (based on Kaplan-Meier curves).

### Is the MiCK assay just a prognostic variable?

In order to test if the maximum degree of apoptosis was prognostic of overall survival without considering whether the patient received the best or a non-best chemotherapy, a Cox proportional hazards model was performed. In all patients receiving chemotherapy, apoptosis was not correlated to survival (p = 0.40). In patients with stage III or IV primary disease, apoptosis was not correlated to survival (p = 0.24). Survival was only correlated with apoptosis if the physician used the best chemotherapy regimen from the MiCK assay (Figure 1).

## Discussion

This prospective, multi-institutional blinded study demonstrated a significant correlation between using the best chemotherapy regimen as assessed in the MiCK assay and overall survival both in all ovarian cancer patients studied, and in the more homogeneous subset of patients with chemotherapy-naïve stage III or IV primary disease. Using the best chemotherapy regimen based on the MiCK assay also correlated with relapse-free interval. If the physicians used a chemotherapy regimen with higher activity in the MiCK assay, response to therapy was higher. This suggests in this non-randomized observational trial that the MiCK assay can help guide selection of more active chemotherapy in ovarian cancer patients. Based on this hypothesis generating study, subsequent randomized validation trials will help further elucidate the benefits of using the MiCK assay to select appropriate therapy for these patients. This study justifies such a randomized trial, and quantifies the benefits in outcomes on which a randomized study can be developed (for power determinations and study size requirements). Such a randomized prospective trial should compare standard postoperative therapy of patients with stages III and IV epithelial ovarian cancer, versus therapy directed by the best results in the MiCK assay. Appropriate stratifications would include extent of debulking, amount of residual disease, stage, age, and preoperative CA125. Numbers of patients needed to treat would be determined by participating statisticians based on primary and secondary goals.

A review by the American Society of Clinical Oncology (ASCO, 1) has found prior chemosensitivity and chemoresistance assays to be insufficiently robust in predicting outcomes, and has not recommended their routine use. This MiCK assay was not reviewed in any of those analyses. However, the reviewers stated in their conclusions that “because the in vitro analytic strategy has potential importance, participation in clinical trials evaluating these technologies remains a priority.” This study represents such a trial that can contribute to subsequent reviews by that ASCO committee and other organizations. This study also represents evidence on which future validation and utility trials can be based.

In contrast to prior chemotherapy resistance assays, the MiCK assay is different. The MiCK assay measures direct in vitro cell killing (rather than measuring only survival of cells), studies only cancer cells (compared to mixed host and cancer cells), requires no in vitro growth of tumor cells (compared to assays requiring growth of cells, which measures only a subset of cancer cells capable of growth in vitro), gives results based on multipoint analyses (every 5 minutes for 48 hours) rather than just a single endpoint [17], and has results available within 72 hours of biopsy (rather than 3–4 weeks).

The ability to identify drugs which are active in the clinical therapy of ovarian cancer suggests that this assay may also be able to play a role in drug development. The assay might be used to study new drugs pre-clinically (2 such trials have been completed, unpublished data), to evaluate if drugs known to be active in other cancers may also have activity in ovarian cancer, and to identify which patients are most likely to respond in clinical trials. This would allow phase II and phase III trials to be focused on patients with the highest chance of improvement, reducing time needed to conduct a clinical trial and increasing the probability of finding effective new drugs. The MiCK assay may also be able to give pharmaceutical companies indications of which approved drugs may be more effectively combined with investigational agents, thus prioritizing clinical trials for more rapid approval by regulatory agencies.

The assay also showed that in some patients (approximately 1/3 to 1/2), single agents were as effective in killing ovarian cancer tumor cells in vitro as standard combination therapies. If confirmed clinically, the MiCK assay may be useful to physicians in individualizing therapy, especially in patients at increased risk of toxicity from combination drug treatments or patients fearful of side effects of combination therapy. Single agent chemotherapy has been found equivalent to combination chemotherapy in some trials [18,19] but not others [20,21].

Since the MiCK assay indicates which drugs are associated with improved survival, it is possible that use of the assay may reduce healthcare costs by avoiding inactive therapies. Results of studies [22] have been used to model potential cost savings in care of cancer patients in a large self-insured employer database. This study indicated possible cost savings of 25 to 85% of chemotherapy-associated expenses.

Since this study initially had a high rate of unsuccessful assays, education of participating sites about submission and processing procedures have been improved which resulted in consistently higher success rates over 75%. Although the learning curve is very fast, the company which provides the test has improved its support of centers using the assay so that inevaluable specimens are less frequent.

In summary, use of the best chemotherapy regimen as assessed in the drug-induced apoptosis MiCK assay correlated with overall survival in all ovarian cancer patients, and in patients with chemotherapy-naïve stage III or IV primary therapy patients with a hazard ratio for death of 0.23. The assay is predictive of survival if physicians use the best treatment based on the assay. Relapse-free interval and response rate were also predicted by the MiCK assay. Further studies of this predictive theranostic bioassay are warranted.

## Misc

Supported by DiaTech Oncology, Nashville, TN.

## Competing interests

The authors declare that they have no competing interests.

## Authors’ contributions

ES participated in design, conducted final reviews and drafted manuscript; MP participated in design and conception of study; HH, MB, AG submitted patients and participated in coordination and review of study results; CP participated in the design and review and manuscript preparation; JR,MC, performed statistical analysis; AH, MP supervised lab results and provided patient outcome data. All authors reviewed and approved the final manuscript.
